# The weight of harm: A Response to “Editor’s Note: Societal changes and expression of concern about Rekers and Lovaas’ (1974) Behavioral Treatment of Deviant Sex-Role Behaviors in a Male Child”

**DOI:** 10.1007/s40617-022-00683-y

**Published:** 2022-03-31

**Authors:** Austin H. Johnson

**Affiliations:** School of Education, University of California, Riverside, Riverside, CA USA

**Keywords:** Behaviorism, Applied behavior analysis, LGBTQ, Retraction, Ethics

## Abstract

In 1974, Rekers and Lovaas published an article in the *Journal of Applied Behavior Analysis* (JABA) wherein the authors coached a 4-year-old child’s parents to ignore and physically abuse him when he engaged in behaviors that were identified by the authors as inappropriate for a child whose sex assigned at birth was male. In October 2020, a Statement of Concern regarding Rekers and Lovaas (1974) was published in JABA (SEAB & LeBlanc, 2020), which described concerns regarding the paper and then provided justification for the journal’s decision to not retract this paper. In this current response, I provide a counterpoint to the Statement of Concern, arguing that (a) the available evidence strongly suggests that the original study was unethical and misaligned with the principles of Applied Behavior Analysis (ABA), and (b) the evidence presented to support its contemporaneous ethicality is insufficient. I end with an argument that Rekers and Lovaas (1974) should be retracted and discuss the critical role of ethics and social significance for the field of ABA.

## Introduction

History is replete with examples of unethical scientific conduct. In the United States Public Health Service Study at Tuskegee, government officials observed the course of syphilis infections in 400 African-American men, did not inform them of their diagnoses, and then deliberately withheld treatment from these men for over 25 years (Paul & Brookes, 2015). Starting in 1955 and 1956, children with intellectual disability were deliberately infected with hepatitis at the Willowbrook State School in a stated effort to “conduct well-designed studies to shed new light on the natural history and prevention of the disease” and the development of a vaccine (Krugman, 1986, p. 159). Beginning in 1990, researchers from Arizona State University collected blood samples from members of the Havasupai (or Havasu Baaja) Tribe under the pretense of conducting diabetes research; after being explicitly told by members of the Tribe that they would not support other uses of their samples for purposes including schizophrenia research, and without any such language being included in consent documentation, these blood samples were used to conduct schizophrenia research (Drabiak-Syed, 2010).

A central defining feature of Applied Behavior Analytic work is the degree to which it examines behavior and stimuli deemed to be important to the individual who is exhibiting that behavior and interacting with those stimuli, as well as the importance of the problem of focus to society as a whole. This central principle was the first of seven articulated by Baer et al. (1968) when they wrote that “a primary question in the evaluation of applied research is: How immediately important is this behavior or these stimuli to this subject?” (p. 93). Individuals who are affiliated with Applied Behavior Analysis (ABA) continue to interrogate how this principle operates in the field, as the ABA literature continually expands to more critically consider how the field intersects with racism (Matsuda et al., 2020), multilingualism (Wang et al., 2019), intersectional identities (DeFelice & Diller, 2019), and the rights and dignity of all people. Given this framework for the field, it is necessary that practitioners who utilize principles from ABA are able to critically examine the research that they consume and the practices that they implement for the degree to which such work fulfills this central tenet of social significance. If a publication that a practitioner consumes or a practice that they engage in does not fulfill this tenet, then in order to meaningfully position themselves within the field, practitioners should be able to recognize that social significance is not taking place, understand why, and identify possible remedies they can take to ensure that it does.

Within scientific publishing, one possible remedy for a research paper that engages in scientific misconduct or other similar practices is an action or event referred to as “retraction” from its publishing journal. Given that science constitutes a wide range of disciplines and massive number of journals, organizations like the Committee on Publication Ethics (COPE) and the Council of Science Editors (CSE) exist to, in part, provide cross-disciplinary guidance on publication ethics and practices, which includes definitions and guidance on retraction procedures. As defined by COPE, retraction involves an official statement from a journal that communicates that the published paper contains “such seriously flawed or erroneous content or data that their findings and conclusions cannot be relied upon,” for reasons which may include fabrication, plagiarism, or unethical conduct (COPE Council, 2019, p. 4). The CSE refers more broadly to “scientific misconduct,” which the CSE defines as “any action that involves mistreatment of research subjects or purposeful manipulation of the scientific record such that it no longer reflects observed truth,” as one possible reason for retraction (CSE Editorial Policy Committee, 2018, p. 47).

As described by COPE, only in rare instances of retraction should the original paper in question be removed completely from the journal’s online presence; however, retraction should always involve an official statement from the journal in print and online formats that the paper has been retracted (2019). This statement should be associated with the online paper itself, and journals should work to ensure that notice of retraction is communicated across bibliographic databases and other online searches. Other indicators can be added to ensure that the reader understands the retracted status of the paper.

The Retraction Watch Database, which is a comprehensive database of papers subject to retractions, statements of concern, or corrections, provides a number of categories for retraction rationale that fall under the umbrella of rectifying such misconduct, including (a) ethical violations, (b) bias issues or lack of balance, and (c) a rationale that they call “doing the right thing” (Center for Scientific Integrity, 2018). As of 10 December 2021, 45 research articles in psychology or education had been retracted for one or more of these three rationales. One such paper, by Glover (1951), was retracted by the *Journal of Nervous and Mental Disease* in December 2020; Glover’s case study of 52 gay men concluded that these men possessed a “narcissistic selfishness in their disregard for people as a whole, no nationalistic or patriotic feeling, a general disdain of inheritance and social values of law, religion and the betterment of mankind” (p. 382). Acting in response to a letter from researcher Simon LeVay, the journal’s editor John Talbott wrote that “the 1951 Glover article supports long discredited beliefs, prejudices, and practices (e.g., conversion therapy) and will be retracted as requested…the 1951 Glover article is but one that deserves a relook, reappraisal, and perhaps retraction” (Talbott, 2020, p. 915). Since being retracted, Glover’s (1951) paper remains accessible on the journal’s website, but the title now reads “Observations on homosexuality among university students [RETRACTED],” and the word “Retracted” appears in large print on the top left of each page of the accompanying PDF, which is aligned with recommendations articulated by the CSE (2018). An editorial from LeVay was also published in the journal alongside the notice of retraction from the journal’s editor in chief.

If a journal does not feel that a retraction is justified or warranted, or if a final decision regarding retraction is pending based upon the findings of a formal investigation, then a journal may instead issue an Expression of Concern (or Statement of Concern), which “is a publication notice that is generally made by an editor to draw attention to possible problems, but it does not go so far as to retract or correct an article” (CSE Editorial Policy Committee, 2018, p. 70). These published statements vary in content and any subsequent action on the part of the journal, but can include electronic notes accompanying manuscript entries that direct readers to the Statement of Concern.

### Rekers and Lovaas (1974)

In October 2020, the *Journal of Applied Behavior Analysis* (JABA) published a Statement of Concern regarding an article by Rekers and Lovaas (1974) entitled “Behavioral Treatment of Deviant Sex-Role Behaviors in a Male Child” (SEAB & LeBlanc, 2020). The Statement of Concern took the form of a manuscript that was written and published in response to concerns brought to the Society for the Experimental Analysis of Behavior (SEAB) and JABA regarding the ethics of the conduct described in the 1974 paper. Additionally, a link to the Statement of Concern was placed on the journal’s web page for the Rekers and Lovaas (1974) paper, such that the existence and content of the Statement of Concern would be visible and accessible to those who reached the journal website for that original manuscript.

Rekers and Lovaas (1974) began their article by enumerating the ways in which their client, a 4-year-old boy identified as “Kraig,” engaged in a number of “feminine” behaviors. In describing this child, whose real name was Kirk Andrew Murphy,^1^ the authors emphasized the extreme perceived pathology of Kirk’s behavior, noting that he “appeared to be very skilled at manipulating [his mother] to satisfy his feminine interests” (p. 174). The authors justified Kirk’s pathologization with four points: (a) feminine behavior by people who are identified by others as male is associated with “social isolation and ridicule”; (b) such behavior is also associated with depression, suicidality, imprisonment, and joblessness; (c) “intervention on deviant sex-role development in childhood may be the only effective manner of treating [i.e., preventing] serious forms of sexual deviance in adulthood”; and (d) these behaviors disturbed Kirk’s parents (p. 175). Although gender identity, gender expression, and sexual orientation are three independent and distinct concepts, Rekers and Lovaas (1974) directly stated that their intervention was aimed to not only reduce Kirk’s feminine behavior, but also to prevent “serious forms of sexual deviance in adulthood” (p. 175); this point was further elaborated by Rekers as being “to prevent transsexualism, transvestism, or homosexuality per se as the most probable adulthood diagnostic outcome in the absence of treatment” (1977, p. 560).

Initial observations were taken by Rekers and Lovaas (1974) of the extent to which Kirk engaged with toys that are socially coded as masculine or feminine in a lab and home setting; the experimental manipulations then began with the researcher prompting an adult accompanying Kirk in a clinic setting to pay attention to and praise Kirk when he played with masculine-associated toys, and ignore Kirk when he played with feminine-associated toys. When Kirk would tantrum in response to his mother ignoring him, the authors reassured her that “she was doing the right thing and was doing it well” (p. 179). At home, Kirk’s mother was first instructed to reinforce Kirk’s behavior with a blue token when he engaged in “nongender behaviors” that were “helpful” and “desired” like washing his hands, and then to punish Kirk’s behavior with a red token when he engaged in either “tantrums and disobedient behaviors” (in the first stage of the study) or non-gender-conforming behaviors (in the second stage; p. 180). These red tokens could result in any of three outcomes for Kirk: (a) losing a point in the token economy earned with a blue token; (b) going into time-out; and (c) being spanked by his father. In Rekers’ (1972) dissertation, upon which Rekers and Lovaas (1974) is based, Rekers noted that at least one prior punishment from Kirk’s father for his feminine behavior had been “severe” (p. 160). Kirk’s brother Mark and sister Maris recalled this period of their lives as exceptionally difficult, with journalist Jim Burroway writing that “Mark today regards the chips as an extremely painful chapter in his life. When I first asked him to describe how they were used, he broke down and sobbed for several minutes, and it took him a long time before he could compose himself…‘I saw my brother’s whole back side bruised so badly one time, my dad should have gone to jail for it’” (2011). In an interview with Anderson Cooper, Kirk’s mother Kaytee recalled similar memories of the punishment, stating that “today, it would be abuse” (Cooper, 2011).

When describing the results of this project, Rekers and Lovaas (1974) noted that they largely observed the behavior changes that they sought, but that Kirk’s masculine behaviors were not generalizing to situations when he was alone; “this, of course, may suggest that he was ‘going underground’ with his deviance, suppressing femininity in the company of adults” (p. 183). They also described the most “effective” form of punishment for Kirk’s behavior:

The disobedient behaviors *did* [emphasis in original] sharply decrease, however, when the red tokens were backed up by spanking. Kraig was told that he would get one “swat” from his father for each red token he collected. After receiving two swats in this manner for red tokens he has received while engaged in nongender-related behaviors, Kraig carefully avoided receiving but a few red tokens from that time on, even though the treatment was to persist for more than half a year (Rekers & Lovaas, 1974, p. 185).

In a section of the paper titled “Informal Clinical Observations,” the authors wrote that “before therapy, Kraig was a ‘crybaby’,” but that after punishing feminine behaviors, “Kraig’s mother began to complain to us that her son had become a ‘rough-neck’…we reassured the mother that such ‘mildly delinquent’ behavior was much easier to correct in future years than feminine behaviors would be” (p. 186). In the discussion section of the paper, the authors described Kirk’s behavior before their actions in the following way:

When we first saw him, the extent of his feminine identification was so profound (his mannerisms, gestures, *fantasies*, *flirtations*, etc., as shown in his ‘swishing’ around the home and clinic, fully dressed as a woman with long dress, wig, nail polish, high screechy voice, *slovenly seductive eyes* [emphasis added]) that it suggests irreversible neurological and biochemical determinants (p. 187).

Upon initial referral to Rekers and Lovaas, Kirk would still have been only 4 years old (Burroway, 2011; Rekers & Lovaas, 1974). Rekers and Lovaas repeatedly stated that Kirk is not the first child that they have engaged in similar actions with, noting that “three observers were already trained in a pilot investigation on normal boys and girls (4 to 7 yr of age) that used identical procedures and materials” (1974, p. 177), and that “we have similar boys in treatment with similar therapy outcomes” whose results are likely to generalize “particularly if these children are quite young (less than 7 yr of age)” (p. 188). This information is confirmed in Rekers’ dissertation (1972), wherein Rekers described “treatment” for five children between 5 and 8 years old who were “the youngest among eight patients available for this research” (p. 54).^2^ The authors noted in the article that they cannot conclude whether “we have produced changes in future preference for sex mates,” but that follow-up data from adolescence and young adulthood “will allow us to claim a preventative treatment for extreme adult deviations of transvestism, transsexualism, or some forms of homosexuality” (p. 188). In the final sentence of their manuscript, the researchers reflected on the future of such “treatments”: “one can entertain some optimism about behavioral treatment of gender role problems, but until more cases are reported, one can only entertain the most tentative hopes that such an effective treatment has been isolated” (p. 188).

Put simply, Rekers and Lovaas (1974) directly facilitated the shaming and abuse of a 4-year-old child because he engaged in behavior that was not judged to be appropriate for his sex assigned at birth. The authors further used disturbing sexualized language (i.e., “fantasies,” “flirtations,” “slovenly, seductive eyes”) when describing this preschool-age child in the corresponding published article.

The term “abuse” has a range of definitions across contexts. The American Psychological Association’s Dictionary of Psychology defines abuse as “interactions in which one person behaves in a cruel, violent, demeaning, or invasive manner toward another person or an animal” (American Psychological Association, n.d.). The intense violence of Kirk’s father’s actions towards Kirk as described by his siblings and mother (Burroway, 2011), the use of the term “severe” in Rekers (1972), and Kirk’s mother’s direct statement that her husband’s beatings constituted “abuse” (Cooper, 2011) suggest that this treatment of Kirk can be accurately described as abuse.

### Statement of Concern

Given these descriptions of Kirk and the actions that were taken to change his behavior, it is unsurprising that members of the ABA community and readers of JABA have expressed concerns regarding Rekers and Lovaas (1974). An official response to these concerns was provided by JABA on 20 October 2020, when JABA published a Statement of Concern (SEAB & LeBlanc, 2020) regarding Rekers and Lovaas (1974). This statement began by acknowledging that early issues of JABA had included articles “that seem controversial in retrospect” (SEAB & LeBlanc, 2020, p. 1830). Providing examples of terms like the r-word, “deviant,” and “mentally handicapped,” the authors noted that terminology has changed over the last decades, just as the use of punishment procedures has changed (p. 1830).

#### Decision to Not Retract

The authors of the 2020 Statement of Concern directly identified its genesis as concerns brought to SEAB and JABA by readers of JABA, and note that SEAB and JABA had been presented with the decision of whether to take action (e.g., retraction) regarding Rekers and Lovaas (1974). The authors of the Statement of Concern wrote that a decision of retraction should be based on one of three major violations according to the Retraction Guidelines provided by COPE (2019). The statement’s authors noted that one such violation is a “clear ethics violation” (SEAB & LeBlanc, 2020, p. 1832). They then stated the following:

By today’s standards and in light of our current scientific knowledge, the study would be considered unethical and would not be published in JABA. However, *the available evidence does not make it clear that the original study was unethical by the standards of that day* [emphasis added]. While the evidential criteria were not met for retraction, SEAB and the Editor of JABA made the decision to issue an official Expression of Concern (COPE, 2019) along with this editorial to clearly outline the concerns about the Rekers and Lovaas (1974) paper and the various harms potentially or actually resulting from it (pp. 1832-1833).

The Statement of Concern (2020) noted that the publication of Rekers and Lovaas (1974) was followed by the submission of two manuscripts in 1975 expressing concern about the ethics and social significance of Rekers and Lovaas (1974), with ultimate publication of these two critical articles in a 1977 issue of the journal (i.e., Nordyke et al., 1977,3; Winkler, 1977). The Statement noted that these critiques articulated concerns with the rationale for intervention, its relevance to contemporaneous discussions of LGBTQ+ (lesbian, gay, bisexual, transgender, queer or questioning, and others) issues, and its ethics, with multiple paragraphs of the Statement describing the content of these two critiques.

Ultimately, however, the Statement noted that there was not sufficient evidence to conclude that the actions and rationale for those actions described in Rekers and Lovaas (1974) were unethical at the time of publication. In support of an argument that the manuscript was unethical, the Statement of Concern included a substantial description of the two critiques that were published in 1977, with specific attention paid to the assertions of Winkler (1977) and Nordyke et al. (1977) that Kirk “was not presenting with distress” (p. 1831). The evidence provided in the Statement of Concern to support an argument that Rekers and Lovaas (1974) was ethical consisted of (a) the project’s federal funding and (b) alignment with the DSM’s pathologization of homosexuality and gender expressions that varied from a person’s sex assigned at birth. The Statement of Concern also noted the nonexistence of the Belmont Report and modern Institutional Review Boards, although as the Statement articulated, “the expectations for considerations of personal rights of participants and use of less intrusive procedures as initial treatment options were also heavily discussed in the scientific community at that time” (p. 1832).

### Critique of the Statement of Concern

In this response, I critique the decision to not retract Rekers and Lovaas (1974) as articulated within the Statement of Concern (SEAB & LeBlanc, 2020). I agree with the Statement of Concern that that the evidence for this paper’s unethical nature is indeed substantial. When viewed alongside the evidence for its ethicality, I argue that it is reasonable to conclude that this manuscript was unethical in 1974. I begin by describing the specific content of the two critiques of Rekers and Lovaas (1974), particularly underscoring the importance of a point that went unaddressed in the Statement of Concern: Donald Baer, the lead author of the field’s document of foundational principles (i.e., Baer et al., 1968), co-authored one of the two published critiques of Rekers and Lovaas (1974) and spoke directly to its absence of social significance. I also describe the corresponding rejoinder by Rekers (1977) that was published alongside both critiques, which was cited but not described in the Statement of Concern. That 1977 rejoinder provided further details regarding Rekers’ rejection of considering the importance of behavior change to the client, and elaborated upon the moral and religious convictions that Rekers (1977) cited as rationale for the actions described in Rekers and Lovaas (1974). I emphasize the connections between the two published critiques and the importance of social significance in ABA, and suggest that such an emphasis in a response co-authored by Donald Baer provides persuasive evidence regarding this foundational figure’s interpretation of the ethical nature of Rekers and Lovaas (1974). I then review the evidence for contemporaneous ethicality forwarded in the Statement of Concern, and challenge both of the main points made in the Statement of Concern supporting the ethical nature of the study. Finally, I end with a reflection on the implications of decisions which privilege the maintenance of research literature when competing and persuasive evidence is provided regarding unethical conduct.

#### Responses from Winkler (1977), Nordyke et al. (1977), and then Rekers (1977)

The contemporaneous response to Rekers and Lovaas (1974) from within the ABA research community was substantial, as two critiques were submitted immediately following its publication in 1975 and then published in 1977 (see SEAB & LeBlanc, 2020, p. 1832). The set of five authors across these two articles (Nordyke et al., 1977; Winkler, 1977), one of whom is recognized as one of the most foundational figures in ABA (i.e., Donald Baer), provided emphatic criticisms of the paper as a whole, and in the case of Nordyke et al. (1977), a point-by-point response to the four rationales used in Rekers and Lovaas (1974) to justify their actions.

Nordyke et al. (1977) began their written response by asserting that Rekers and Lovaas’ (1974) paper is “not only accepting but also supporting sex-role stereotyping, thereby failing to contribute to the solution of a larger social problem” (p. 553). This first sentence of the response co-authored by Baer reflects the description of the first guiding principle of ABA proposed by Baer and colleagues in 1968: the applied nature of ABA “is not determined by the research procedures used but rather by the interest which society shows in the problems being studied” (Baer et al., 1968, p. 92). Nordyke and colleagues then described why the four rationales provided in Rekers and Lovaas (1974) were not sufficient for demonstrating that their work with Kirk was socially significant, stating that “not every social pressure, not even every extensive social pressure, need be taken to define a deviancy that thereby needs treatment” (1977, p. 554). Similarly, in his response to the Rekers and Lovaas paper, Winkler (1977) wrote that “where ‘pathology’ is associated with sexual deviance, much of it, if not all, can be regarded as a function of social attitudes to sexual behavior” (p. 550).

Rekers was provided with the opportunity to respond to these criticisms with a rejoinder, which was published in the same issue of JABA in 1977 as the two criticisms. In this rejoinder, Rekers updated his total number of justifications for engaging in conversion therapy with Kirk from four to eight, and throughout the rejoinder, Rekers was transparent in describing that his religious convictions and personal opinions of people who are LGBTQ were a driving force behind his research, as “parents may reject dishonesty or homosexual behaviors as wrong on moral grounds, regardless of what percentage of the population happily engages in those behaviors” (1977, p. 560). Indeed, the seventh of Rekers’ eight listed justifications in 1977 for engaging in behavior modification with Kirk was that “the intervention goals were consistent with the Christian ethical value system (see Evans, 1975) held in common by the parents and the therapist, Rekers” (p. 564).

In his rejoinder, Rekers went on to state that the two critical 1977 articles provided no evidence that “most parents, if given a choice, would consider it desirable to foster homosexuality, transsexualism, or transvestism in their child” (1977, p. 560). Rekers further emphasized the ultimate irrelevance of the client’s happiness in justifying intervention when he stated that “a parent could legitimately request the prevention of homosexual behavior, for example, on the basis that it is morally wrong, even if it were possible for the child to develop as a contented homosexual” (p. 563). Indeed, Rekers repeatedly asserted that the parents’ interests in this case were more important than those of Kirk, going so far as to state that “by itself, the child’s lack of choice in an intervention does not pose any legal or ethical problem” (1977, p. 564).

In response to Winkler’s (1977) assertion that the professionally and ethically correct decision would be to support Kirk’s identity, Rekers wrote in his rejoinder that “we find this line of argument to be ethically unacceptable (Evans, 1975) and professionally irresponsible” (1977, pp. 565-566), supporting his assertion with the notion that “there are specific behaviors that are inappropriate for males in all circumstances…it is an important socialization process for the *boy* [emphasis in original] to learn that he will not grow up with the biological possibility of having sexual intercourse with a man” (p. 566). Rekers (1977) was continuously and emphatically assertive in writing that “this kind of debilitating sex-role inflexibility” is unethical, immoral, and must be changed (p. 566); quite simply, “nurturant behavior in a boy is desirable, but when that behavior is accompanied by verbalizations of a female identity, it is undesirable” (p. 567). In a direct statement regarding gender conversion therapy, he wrote that “improved general social adjustment and peer relationships have been reported for gender-disturbed boys who have made such a transition with intervention” (p. 567), ending his rejoinder with the assertion that this was “an intervention that was ethically and psychologically appropriate” (p. 569). Although this rejoinder is richly illustrative of Rekers’ (1977) motivations for engaging in the study, it was not described in the Statement of Concern (SEAB & LeBlanc, 2020).

For their part, Nordyke et al. (1977) closed their response to Rekers and Lovaas’ original 1974 article with the following statement: “we question the methods that appear to be the result of the researchers’ own sex-role stereotyping. Only time and monitoring will tell the outcomes” (p. 557).

Kirk Murphy committed suicide in 2003.

#### Abundant Evidence of Contemporaneous Ethical Concerns

The Statement of Concern (SEAB & LeBlanc, 2020) contained substantial descriptions of the contemporaneous criticisms by Winkler (1977) and Nordyke et al. (1977). In these criticisms, Winkler (1977) and Nordyke et al. (1977) clearly stated that the actions taken by Rekers and Lovaas (1974) did not conform to their understanding of the standards of the field, which had been co-written by one of these responses’ co-authors. Nine years earlier, Baer and colleagues had described ABA as being rooted in a standard of socially significant behavior change as evaluated using two critical tests: whether the target behaviors were selected “because of their importance to man and society, rather than their importance to theory,” and “how immediately important is this behavior or these stimuli to this subject?” (1968, pp. 92-93). Nordyke et al. (1977) wrote that Rekers and Lovaas (1974) failed this test, and in response, Rekers (1977) rejected this standard altogether, stating that the importance of behavior change centered upon Kirk as an individual is fundamentally irrelevant; indeed, intervention would be warranted for Kirk “even if it were possible for the child to develop as a contented homosexual” (p. 563).

This evidence would appear to strongly suggest that this research would be considered to be outside the bounds of acceptable practice in ABA in 1974. The two described critiques were submitted the year immediately following the publication of Rekers and Lovaas (1974; see Statement of Concern, 2020). Both critiques spoke directly to the researchers’ assumptions, rationale, practices, and awareness of contemporary issues in LGBTQ rights. In one of them, the lead author of the foundational principles of ABA stated unambiguously that Rekers and Lovaas (1974) did not meet one of those foundational principles.

Additionally, although unaddressed in the Statement of Concern (SEAB & LeBlanc, 2020), the language used in Rekers and Lovaas (1974) to describe Kirk Murphy is disturbing. The use of a sexualized constellation of words that describe a 4-year-old child as having “fantasies,” “flirtations,” and “slovenly seductive eyes” is unambiguously concerning, particularly when viewed alongside the evidence for unethical conduct already presented (1974, p. 187).

### Statement of Concern’s Evidence for Ethical Conduct

The Statement of Concern did not describe what specific information or balance of evidence would have been sufficient to meet retraction criteria, only that “the evidential criteria were not met” (SEAB & LeBlanc, 2020, p. 1832). In reviewing the Statement of Concern, the Statement relied on two central arguments to support an assertion that this research could be considered ethical in the early 1970s: (a) the American Psychiatric Association (APA), the Diagnostic and Statistical Manual (DSM), and others still considered homosexuality and gender expressions different from a person’s sex assigned at birth to be pathological, and (b) the research was federally funded.

#### Diagnostic and Statistical Manual (DSM), Homosexuality, and Contemporaneous Attitudes

It is not entirely accurate to state that APA and the DSM still considered homosexuality to be pathological in 1974; homosexuality was removed as a psychopathology from the DSM in 1973 after sustained activism from members of the LGBTQ community (Drescher, 2015). This decision was supported by 58% of 10,000 voting APA members when it was placed before them in response to concerns raised by psychoanalysts about the removal. The years leading up to this decision were replete with LGBTQ activism in the psychological sphere; in 1970, LGBTQ activists disrupted a paper presentation at APA’s Annual Meeting by Nathaniel McConaghy “who was discussing the use of aversive conditioning techniques in the treatment of sexual deviation” (Bayer, 1987, p. 103). As described by Bayer, the paper presentation itself was consistently interrupted by activists shouting “vicious” and “torture,” with the resulting disruption being so significant that some psychiatrists demanded that APA refund their airline tickets. Subsequent panels were convened to discuss “the stigma caused by the ‘homosexuality’ diagnosis” at both the 1971 and 1972 meetings of APA (Drescher, 2015, p. 570).

Such discussions were also clearly present within ABA as well. In his critique submitted to JABA in 1975, Winkler wrote that “there is now considerable, replicated evidence from surveys of nonpatient homosexuals that homosexuals are not more abnormal or less well-adjusted than heterosexuals,” and went on to point out that Rekers and Lovaas “make no mention of the evidence of the changing attitudes to homosexuality and other sexual behavior labelled deviant, evidence such as changing laws, gay liberation movements, and psychiatric opinion” (1975, p. 550). Attitudes towards people who are LGBTQ were not monolithic in 1974 in society, psychology, or ABA. Indeed, members of the ABA community viewed what Rekers and Lovaas did as in fundamental opposition to the stated mission and principles of the field.

The Statement of Concern (2020) acknowledged these tensions and stated that homophobic and transphobic attitudes were not uniform, although some “clearly felt that homosexuality was pathological” (p. 1831). To support this claim, the Statement of Concern parenthetically cited two papers. The first was a manuscript by Bieber (1976), wherein he asserted that adolescents are “particularly vulnerable to homophile propaganda and misinformation. The notion that homosexuality is normal and should not be treated only reinforces denial and resistance” (p. 166). The second was Rekers’ own 1977 rejoinder.

Nearly 50 years later, statements like those made by Bieber (1976) and Rekers (1977) remain easy to find, and it is likely naïve to assume that such prejudice will ever disappear completely. Hate may always exist, but it is unclear why the existence of such prejudice also legitimizes the ethics of actions that restrict the health and well-being of LGBTQ people.

#### Federal funding for Rekers and Lovaas (1974)

Despite the harm of such research, the federal government did fund this study and other studies like it. The NIMH gave O. Ivar Lovaas $54,200 to engage in gender conversion therapy with children whose gender presentations differed from their sex assigned at birth for the grant acknowledged in Rekers and Lovaas (1974). As Nordyke and colleagues noted in their critical response: “clearly the experimenters’ ideas about sex-role are also shared by others. Indeed, the study was supported by a research grant from the National Institute of Mental Health” (1977, p. 556).

However, it is not hard to find examples of projects that were federally funded but also recognized as unethical during the time of their funding. The United States Public Health Service Study at Tuskegee was a federally funded project that was initiated in 1932 and *still taking place* in 1972 when the Tuskegee Syphilis Study Ad Hoc Advisory Panel determined that “the scientific merits of the Tuskegee Study are vastly overshadowed by the violation of basic ethical principles pertaining to human dignity and human life imposed on the experimental subjects” (p. 11). In 1963, Chester Southam and Emanuel Mandel injected live cancer cells into patients in a study funded by the United States Public Health Service and the American Cancer Society. They were found guilty of research misconduct by their university in 1964, and in 1965, National Institute of Health official Joseph Murtaugh stated regarding this case that “it made us all aware of the inadequacy of our guidelines and procedures…the judgment of the investigator is not sufficient as a basis for reaching a conclusion concerning the ethical and moral set of questions in [the relationship between patient and experimenter]” (Committee on Labor and Public Welfare, 1971, p. 226). Quite simply, given the standards for research conduct and oversight in the mid-twentieth-century, it is erroneous to equate federal funding with contemporaneously acceptable judgments of research ethics.

### Implications and Future Directions

As presented in the 2020 Statement of Concern (SEAB & LeBlanc), there is evidence to support both sides of the question of contemporaneous ethicality of the actions described in Rekers and Lovaas (1974). I view the evidence of unethical conduct described in this current critique to be substantially stronger than that presented in the Statement of Concern for its ethicality (SEAB & LeBlanc, 2020). In addition, it is important to reiterate the fundamental wrongness of research that is willing to ascribe sexual intent to a 4-year-old, as Rekers and Lovaas detailed this preschool-aged child’s “fantasies,” “flirtations,” and his “slovenly seductive eyes” (1974, p. 187). I have argued that the evidence is substantial to support a conclusion that Rekers and Lovaas (1974) meets the Statement of Concern’s (2020) cited criterion for retraction as articulated by COPE: “it reports unethical research” (2019). As stated in the 2020 Statement of Concern, JABA determined that this criterion was not met. I therefore end with a reflection on the significance of this decision.

First, I believe that it is an error to employ a heuristic that, when faced with compelling evidence that harm was done to a child and research participant, nonetheless rejects a conclusion that unethical conduct took place due to the presence of evidence that some considered such conduct to be ethical at the time of publication. The evidence available regarding what happened to Kirk Murphy clearly demonstrates that Rekers and Lovaas caused harm to a child, and that the principles used to justify it were contrary to the core principles of ABA as defined in 1968. Indeed, this research’s opposition to these core principles was recognized and articulated by foundational members of this field at the time of that article’s publication.

If an argument is forwarded that such research deserves an equal place in the scientific canon because it is a legitimate demonstration of behavioral principles, then I believe that it is worth revisiting Baer, Wolf, and Risley when they defined the difference between the “applied” and the “non-applied” researcher in 1968: “in behavioral application, the behavior, stimuli, and/or organism under study are chosen because of their importance to man and society, rather than their importance to theory” (p. 92). In articulating the principles for the field, these authors were clear that ABA is defined by this “applied” use of behaviorism; research that has “importance to theory” but lacks “importance to man and society” is not an application of ABA.

Further, if concerns about censorship or revisionism with respect to retraction are present, I would revisit the purpose of retraction as articulated by COPE: “to correct the literature and ensure its integrity” (2019). The organization goes on to write that “retractions may be used to *alert readers* to cases of redundant publication, plagiarism, peer review, manipulation, reuse of material or data without authorisation, copyright infringement or some other legal issue (e.g., libel, privacy, illegality), *unethical research*, and/or a failure to disclose a major competing interest that would have unduly influenced interpretations or recommendations [emphasis added]” (2019). The publication process is complex, and across science, journals are regularly faced with decisions of determining whether material that has been given the recognition of the scientific canon in the past represents conduct that warrants such a distinction. To that end, retraction has been clearly defined within scientific publishing as a mechanism for alerting the reader to unethical research. Scientific publishing remains the de facto mechanism through which knowledge is evaluated and disseminated in the sciences, and as such, mechanisms that exist to ensure its integrity should be implemented clearly and consistently.

Unethical research compromises the integrity of the scientific literature that researchers and practitioners who utilize the principles of ABA to make socially significant differences in individuals’ lives depend upon to inform their practice. It is therefore imperative that researchers and practitioners in ABA examine whether the science that they depend upon is socially significant and ethical. Intentionally facilitating the harm of a child because of their gender expression, and then describing that child in sexualized and developmentally inappropriate terms, is neither.

## Footnotes

1 Based on conversations with Kirk’s sister, Maris, Kirk’s pronouns were he/him and he identified as a cisgender gay man; thus, I use these pronouns when referring to Kirk (M. Ehlers, personal communication, November 11, 2020).

2 Kirk is also described in Rekers’ 1972 dissertation, but he is referred to as 5 years old, not 4 years old, likely because he was 4 years 11 months (i.e., nearly 5 years old) at the time of referral to Rekers and Lovaas.

3 Please note that the “LeBlanc” authors cited in (a) Nordyke et al. (1977) and (b) SEAB and LeBlanc (2020) are two different people. The co-author of Nordyke et al. (1977) is Judith M. LeBlanc. The co-author of SEAB and LeBlanc (2020) is Linda A. LeBlanc.
