# Correction to: The weight of harm: A Response to “Editor’s Note: Societal changes and expression of concern about Rekers and Lovaas’ (1974) Behavioral Treatment of Deviant Sex-Role Behaviors in a Male Child”

**DOI:** 10.1007/s40617-022-00711-x

**Published:** 2022-04-19

**Authors:** Austin H. Johnson

**Affiliations:** School of Education, University of California, Riverside, Riverside, CA USA

**Correction to: Behavior Analysis in Practice**

 10.1007/s40617-022-00683-y

This article was updated to correct a reference citation and to add a reference.

